# Protective sexual behaviours among young adults in Nigeria: influence of family support and living with both parents

**DOI:** 10.1186/s12889-019-7310-3

**Published:** 2019-07-23

**Authors:** Anthony Idowu Ajayi, Sylvester Reuben Okeke

**Affiliations:** 1 Population Dynamics and Reproductive Health Unit, African Population and Health Research Center, APHRC Campus, Nairobi, Kenya; 2 Centre for Social Research in Health, University of New South Wales, Sydney, Australia

**Keywords:** Sexually transmissible infections, Adolescents, Risky sexual behaviour, Abstinence, Sexual fidelity, Consistent condom use, Behaviour change interventions, Sub-Saharan Africa

## Abstract

**Background:**

Many studies have focused on risky sexual behaviour among adolescents and young adults; however, literature on protective sexual practices among this age cohort is still evolving. Since young adults are disproportionately burdened by sexually transmissible infections, including HIV, understanding factors that influence protective sexual behaviour among the age group is crucial in developing age-appropriate interventions. Drawing from a cross-sectional survey conducted among adolescents and young adults in two Nigerian universities, we examined gender differences in protective sexual behaviours and the influence of family support and living with both parents on these behaviours.

**Methods:**

A total of 800 male and female university students in two Nigerian universities were recruited using stratified random sampling between February and April 2018. Analysis was, however, based on 599 participants aged between 15 and 24 . Adjusted and unadjusted multinomial logistic regression models were used to examine the influence of family support, and living with both parents on protective sexual behaviours at a 95% confidence interval.

**Results:**

Findings show that the largest proportion of our participants engaged in protective sexual behaviours. We found no gender differences in protective sexual behaviours, including sexual abstinence, consistent condom use, and sexual fidelity. Family support and living with both parents were positively associated with protective sexual behaviours among adolescents and young adults.

**Conclusion:**

This study found that a majority of adolescents and young adults in Nigerian Universities engage in protective sexual behaviours. Adequate family support and living with both parents are positively associated with protective sexual behaviours. The study however revealed that about one-fifth of our participants engaged in high-risk sexual behaviour. This suggests a need for behavioural change interventions, provision of sexual health services and empowerment of students who receive inadequate family support.

## Background

Protective sexual behaviour is conceptualized in this study as any behaviours targeted at preventing sexually transmissible infections (STIs) or unplanned pregnancies by sexually active or inactive individuals. These behaviours include abstinence from sex, sexual fidelity and consistent condom use. This paper builds on the work of Cort and Tu [1] who viewed sexual behaviour as a continuum; with individuals who engage in unprotected sex with multiple sexual partners on the higher end of risk continuum, while those who abstain from sex occupy the lower end of the risk continuum.

Most studies on the sexual behaviour of adolescents and young adults tend to characterise condomless sex and multiple sexual relationships as risky sexual behaviour [2–6]. While this is true to some extent, these studies may have overestimated the prevalence of risky sexual behaviours. Sexual fidelity, that is, unprotected sex with one uninfected partner and consistent condom use with multiple sexual partners are often not considered when researchers categorise non-condom use and multiple sexual relationships as high-risk sexual behaviours.

To view risky sexual behaviour – in relation to unplanned pregnancy – as engaging in condomless sex, ignores the fact that people can prevent unplanned pregnancies after sex with the use of emergency contraception or may be using other contraceptive methods. In relation to STIs, it also overlooks the fact that faithfulness to one uninfected sexual partner could prevent such infections. In other words, not all adolescents or young adults who engage in unprotected sex could be said to involve in high-risk sexual behaviour. Also, since consistent and correct use of condoms with multiple sexual partners could mitigate the risk of STIs transmission, not every individual with multiple sexual partners could be classified into a high-risk group. Our view of sexual behaviour as a continuum, thus, departs from the binary view of sexual behaviour, which is common in sexual health literature.

The transition from childhood to adolescence represents a critical stage in the life of a person [7–9]. This phase is characterised by experimental behaviour that could threaten long term health and wellbeing [7–9]. In the Nigerian context, sexuality is viewed as a realm requiring adult maturity; which adolescents lack. Consequently, becoming sexually active as an unmarried adolescent is depicted as abnormal, experimental and risky. This negative portrayal of sexual actieveness at adolescence (and while unmarried) as “abnormal”, creates a culture of silence and restricts open discussion of sexuality issues affecting young people. This restriction, consequently, predisposes adolescents and young adults to adverse outcomes such as high teenage pregnancy, unsafe abortion, disproportionate HIV acquisition and even deaths [10–12].

There is a significant gender difference in the sexual behaviour of young people in sub-Saharan Africa [13–15]. There is also evidence that sexual initiation occurs earlier among females compared to males [14]; although males are more likely to have multiple sexual partners than females [15]. What remains unclear is whether gender difference exists in protective sexual behaviour of adolescents and young adults in this context.

In general, the literature on adolescents and youth sexual behaviours are dominated by studies examining risky sexual behaviours and its determinants [2–6]. However, the practice of protective sexual behaviours and its determinants among this cohort has received little attention. A review of the literature shows that family structure (that is, living with single or both parents or none of them) is associated with the sexual behaviour of young people [16–18]. Other family-related factors like parent-adolescent sexual communication [19–21], parent-child co-residence instability [22] and socio-economic background [23] have been found to also influence the sexual behaviours of young people. However, the influence of living with both parents on protective sexual behaviour is less understood.

Likewise, the influence of family support on protective sexual behaviours among adolescent and young adults is less understood, especially in the Nigerian context. In Nigeria, students depend mainly on their parents to fund their university education as there are no students loans, and scholarship opportunities are few or almost non-existent. A clear understanding of the role of family support and living with both parents on protective sexual behaviour is vital in informing programmes and policies in the continued efforts aimed at reducing STIs and unplanned pregnancy among young people in Nigeria.

The present study draws from a cross-sectional survey conducted among adolescents and young adults in two Nigerian universities to examine the practice of protective sexual behaviours. Also, the study examines whether there is a gender difference in the practice of protective sexual behaviours among the participants. Additionally, we examined the influence of family support and living with both parents on protective sexual behaviours of our adolescents and young adults participants. Previous studies have characterised Nigerian universities as a space devoid of parental monitoring and supervision, thus allowing young people to date freely without hindrances, nurture relationships, engage in sex, transact sex, or otherwise engage in risky sexual behaviours [3, 4, 24–27]. The findings of this study could inform appropriate and research-oriented policies to address the sexual and reproductive health needs of adolescents and young adults – which are currently lacking – in Nigerian higher education institutions.

## Methods

The data analysed in this study were drawn from a cross-sectional survey conducted in two Nigerian universities between February and April 2018. This paper is a part of a larger study that examined the reproductive health and wellbeing of university students in Nigeria. The detailed methodology is published elsewhere [28]. The two universities, one owned by the federal government, and the other by the state government, were selected purposively. One university was selected in Nasarawa state because it is located in a high HIV prevalence area, and the other was chosen in Ilorin because of the plurality of students attending the university. Trained research assistants administered questionnaires to 800 participants through a face-to-face interview in private settings. Participants were selected using stratified sampling. Stratification was based on sex, level of study and faculty of study and probability proportionate to the size of each stratum was selected. The research assistants recruited students into the study by visiting every faculty and recruiting them from their classrooms. The questionnaire was paper-based and was completed mainly in the form of a face-to-face interview, except for the participants that insisted on completing the survey on their own. The response rate was very high, with only a few participants (5%) refusing to participate in the study. No form of compensation was given to respondents for their participation in the study.

To validate the self-design instrument, we conducted a pilot study among 20 participants in another university. The feedback from these 20 participants was used to improve the questionnaire. Data collection took place from February to April 2018. The ethical review committee of the University of Fort Hare and Ondo State Ministry of Health approved the study protocol. All participants provided signed informed consents after confidentiality was guaranteed. Participants were unmarried male and female university students selected using stratified sampling. The sample size was determined using the Epi-info sample size calculator. The required sample size from each university was 400 participants, based on 50% HIV testing uptake previously reported in the study setting, 95% confidence level, +/− 5% precision level and 5% adjustment for possible missing responses. Given that this study focuses on adolescents and young adults, we only performed analysis on 599 respondents who were within the age group 15–24 years.

### Measures

#### Dependent variable

Our main dependent variable is a six nominal category measure of protective sexual behaviours. This measure was derived from a series of questions probing the sexual behaviours of participants. We asked participants whether they have ever engaged in sex, whether they engaged in sex in the last year, and the number of sexual partners they had in the previous year. Also, participants were asked if they used condoms at all sexual encounters in the past year and the type of partners they had sex with. In line with our argument and consistent with Cort and Tu’s [1] operationalisation of the concept, our measure of protective sexual behaviours took into account never engaged in sex, abstinence in the past 1 year, consistent condom use and the practice of sexual fidelity. We created six categories by combining these behaviours in the following ways:

- Category 1 Never engaged in sex
- Category 2 Had sexual experience but abstained from sex in the past year
- Category 3 Non-abstinent- Used condoms consistently and practising sexual fidelity
- Category 4 Non-abstinent- Used condoms consistently and not practising sexual fidelity
- Category 5 Non-abstinent- Don’t use condoms consistently and practising sexual fidelity
- Category 6 Non-abstinent- Don’t use condoms consistently and not practising sexual fidelity

However, we recategorised protective sexual behaviour into three categories because of insufficient sample size to sustain adequate statistical power for all six categories. Both never engaged in sex and abstained from sex in the past year were grouped and renamed sexual abstinence. Category 3 to 5 were grouped and renamed as “safer sex” while the sixth category was renamed as high-risk sexual behaviour.

#### Independent variable

The main independent variables were family support and family structure. Family support was operationalised as whether participants receive adequate, moderate, insufficient and no support from home. In this study, family support refers to economic support received from parents who are considered to as the main sponsors of students tuition fee, accommodation fee, subsistence on campus, and textbook purchase as student loan is not available in the country. Anectodal evidence shows that students depend solely on their parents to pay their fees, which is sometimes difficult for students from indigent families. Participants were asked whether their mother and father are alive and if they currently live with them. Family structure was categorised as living with both parents, living with one parent and not living with both parents.

#### Covariates

We included two sets of covariates: demographic factors and lifestyle behaviour factors. The control variables for demographic factors include age and sex, while the controls for lifestyle behaviours include alcohol use and drug use, which have all been shown to be associated with risky sexual behaviours [29, 30]. Drug use was assessed by asking participants if they have ever used drugs such as cannabis, tramadol, and codeine for pleasure or to ease tension. A follow-up question was asked to probe those who had ever used drugs if they currently use it and whether they used drugs in the past 30 days. Also, alcohol use was assessed with a question probing lifetime use of alcohol and a follow-up question on recency of alcohol use. A binary response of “yes” or “no” was provided for participants to choose in response to the questions on drug and alcohol use. Age was measured as a continuous variable.

#### Data analysis

Data were coded and entered into Statistical Package for the Social Sciences (Version 24) for statistical analysis. All variables of interest were analysed using descriptive statistics. Mean, and standard deviation were used to summarise the continuous variables, while frequency counts and percentages were calculated for categorical variables. To test the study hypotheses, we fitted three multinomial logistic regression models. The first model was a bivariate model, which was used to examine the net effect of each main independent variable on the outcome variable. The second model was a multivariate model which contained the demographics, while the third model included both demographic and lifestyle behaviour controls. All models are fitted at a 95% confidence interval. Alpha values less than 0.05 were considered statistically significant.

## Results

The analysis was performed on 599 participants aged below 25 years. The average age of study participants was 20.42 years (SD = 2.23). The demographic and behavioural characteristics of the study participants are presented in Table 1. Over a third of the participants (36.6%) were still in their teenage years, while 53.9% of them were female.

**Table 1:** Demographic and behavioural characteristics of respondents

Variables	Frequency	Percentage
Age		
15–19 years	219	36.6
20–24 years	380	63.4
Sex		
Male	276	46.1
Female	323	53.9
Father alive		
Yes	522	87.1
No	77	12.9
Live in the same household as your father		
Yes	477	74.6
No	152	25.4
Mother alive		
Yes	547	91.3
No	52	8.7
Live in the same household as your mother		
Yes	500	83.5
No	99	16.5
Family structure		
Single parent	129	21.5
Nuclear family	372	62.1
Polygamous family	69	11.5
Living with foster parents	29	4.8
Family support		
Adequate	445	74.3
Moderate	115	19.2
Insufficient support	27	4.5
No support	12	2.0
Currently drink alcohol		
Yes	159	26.5
No	440	73.5
Currently smoke		
Yes	77	12.9
No	522	87.1
Currently use drug		
Yes	99	16.5
No	500	83.5
Protective Sexual behaviours		
Never engaged in sex	134	22.4
Has sexual experience but abstained from sex in the past one year	109	18.2
Used condoms consistently and practising sexual fidelity	95	15.9
Used condoms consistently and not practising sexual fidelity	56	9.3
Don’t use condom consistently and practising sexual fidelity	94	15.7
Don’t use condom consistently and not practising sexual fidelity	111	18.5

Regarding family characteristics, a majority of the participants’ fathers and mothers are alive, and they currently live with their parents. Most participants were from a nuclear family (62.1%) and received adequate support from home (74.3%). A few participants reported current use of recreational drugs (16.5%), tobacco smoking (12.9%) and alcohol use (26.5%). Slightly over one-fifth of the participants had never engaged in sex, while 18.2% of them abstained from sex in the past year. Overall, 18.5% of the participants engaged in high-risk sexual behaviour defined as inconsistent condom use and not practising sexual fidelity.

### Gender and protective sexual behaviours

As shown in Table 2, there were no gender differences in protective sexual behaviours. Even though males had lower odds of sexual abstinence, the 95% confidence interval for the odds included 1.0, thus indicating a null association between gender and sexual abstinence.

**Table 2:** Multinomial Logistic Regression Model Showing Association Gender and Protective Sexual Behaviour

Protective Sexual Behaviours	Odds Ratio	95% CI
Sexual abstinence (never had sex or abstained from sex in the past one year)	0.88	0.56–1.37
Play safe (used condoms consistently or practicing sexual fidelity	0.97	0.62–1.52

*P < 0.05, *CI* Confidence Interval, The reference category was female

### Family support and protective sexual behaviours

The findings on the relationship between family support and protective sexual behaviour are presented in Table 3. We used Model 1 to establish whether the odds of a particular type of protective sexual behaviour vary by level of family support. The results show that family support is positively associated with protective sexual behaviour. Individuals who received adequate or moderate support from home had a higher likelihood of sexual abstinence compared to those who received no or insufficient support. Also, individuals who received adequate and moderate family support had higher odds of safer sex; the relationship, however, was not statistically significant.

**Table 3:** Multinomial Logistic Regression Models of Protective Sexual Behaviour and Family Support

Variables	Model 1^a^		Model 2^b^		Model 3^c^	
	OR	95% CI	OR	95% CI	OR	95% CI
Sexual abstinence						
Adequate family support	10.35	3.35–32.24***	7.47	2.36–23.63*	5.17	1.52–17.61*
Moderate family support	6.45	1.93–21.59*	5.53	1.64–18.69*	4.16	1.14–15.21*
Safer sex						
Adequate family support	1.88	0.91–3.88	1.91	0.91–4.00	1.45	0.66–3.16
Moderate family support	1.41	0.62–3.22	1.42	0.62–3.25	1.16	0.49–2.78

***P-value< 0.001; *P-value < 0.05; *CI* confidence interval

^a^No covariate variables present

^b^Sociodemographic variables present as covariates

^c^both sociodemographic and lifestlyse behaviours variables were present as covariates

In Model 2, we included sex and age as covariates. Results from this model suggest that adequate family support is associated with a higher likelihood of protective sexual behaviour. After controlling for both sociodemographic and lifestyle behaviour variables in Model 3, the relationship between family support and protective sexual behaviour remain positive and significant, thus establishing the influence of family support on protective sexual behaviour.

### Relationship between living with both parents and protective sexual behaviours

The findings on the relationship between living with both parents and protective sexual behaviours are presented in Table 4. In Model 1, living with both parents was positively related to protective sexual behaviours. Individuals who lived with both of their parents had a higher likelihood of practising sexual abstinence. This finding holds after controlling for age and sex in Model 2. However, the odds for protective sexual behaviour crossed the null value of 1 after controlling demographic variables and lifestyle behaviours variables in Model 3.

**Table 4:** Multinomial Regression Models of the Relationship Between Living With Both Parents and Protective Sexual Behaviours

Variables	Model 1^a^		Model 2^b^		Model 3^c^	
	OR	95% CI	OR	95% CI	OR	95% CI
Sexual abstinence						
Living with both parents	3.75	1.61–8.76*	3.63	1.51–8.73*	1.60	0.60–4.27
Living with either mother or father	3.38	1.35–8.43*	3.32	1.30–8.53*	2.04	0.72–5.75
Play safe						
Living with both parents	1.20	0.94–4.23	2.01	0.94–4.30	1.22	0.54–2.77
Living with either mother or father	2.33	1.03–5.29*	2.34	1.03–5.35*	1.82	0.77–4.32

***P-value< 0.001; *P-value < 0.05; *CI* confidence interval

^a^No covariate variables present

^b^Sociodemographic variables present as covariates

^c^both sociodemographic and lifestlyse behaviours variables were present as covariates

## Discussion

Building on existing research that departs from the binary view of sexual behaviours [1], this study examined the practice of protective sexual behaviours of adolescents and young adults using data obtained from two Nigerian universities. Our analysis shows that most students practised protective sexual behaviours: lifetime abstinence or abstinence in the past year, consistent condom use and sexual fidelity. However, about one-fifth of the students engaged in high-risk sexual behaviour defined as inconsistent condom use and not practising sexual fidelity. Our findings on the sexual behaviours of young adults are more nuanced compared to previous studies [31–33]. We are able to show how young adults are protecting themselves from contracting sexually transmissible infections in our study settings. Also, it has been established that educated young adults are aware of the risk associated with unprotected sex but still engage in such behaviour [34]. Even though the sexual practices characterised as protective in this study may prevent STIs and HIV, the risk of an unplanned pregnancy cannot be ruled out. In other words, sexual fidelity could reduce the risk of HIV and STI transmission, but many of these students are still faced with the risk of unplanned pregnancies and consequently, clandestine abortions, which are associated with complications and even death.

While previous studies show that a large proportion of adolescents and young adults are engaging in high-risk sexual behaviours, our findings suggest that only one-fifth of them could be characterised as such. This relatively low proportion is, however, still too high. There is, therefore, a need for sexual health programme intervention on Nigeria campuses. Providing access to HIV testing, primarily through the provision of HIV self-testing for students, could be an important intervention in the study settings. Studies have shown that people embrace risk reduction behaviour after they tested for HIV [35]. Available evidence indicates that the rate of HIV testing uptake is low among Nigerian university students [36]. Considering the low uptake of HIV testing and high-risk sexual behaviour observed among Nigerian students, sexual health intervention is urgently needed among this cohort. Adolescents and young adults are the potential future leaders of the country; as such, their sexual health and well being should be at the forefront of governmental social policy interventions.

We found no gender differences in protective sexual behaviour, including sexual abstinence and safer sex. Anecdotal evidence suggests that male are more likely to have multiple sexual partners, especially in a patriarchal society like the study setting. This finding could also mean that men tend to have numerous sexual partners but always used condoms with those partners, while women are less likely to insist on condoms with their multiple sexual partners. A study has shown that males are more likely to report condom use at last sex compared to females [13].

This study also found that family support is positively associated with protective sexual behaviours among adolescents and young adults. Individuals who received adequate or moderate support from home had a higher likelihood of reporting not to have engaged in sex compared to those who received insufficient or no support. Similarly, adequate family support significantly increases the possibility of practising sexual abstinence. This association persisted after controlling for demographic factors and also after separately controlling for drug and alcohol use. It is plausible that students engage in sex with multiple sexual partners for benefits in order to augment the insufficient support they receive from home. In other words, high-risk sexual behaviour becomes a survival mechanism to cope with deprivation. Studies have shown that transactional sex is common on Nigerian campuses [37–39]. Transactional sex is disempowering for young girls and often limits their ability to negotiate condom use. Sex for support and benefits is disempowering and increases the risk of HIV transmission, especially among adolescent girls and young women [40, 41]. However, the reason why male students engage in sex with multiple sexual partners without using condom needs further investigation. Adequate family support is protective against risky sexual behaviour and thus suggests that providing funding opportunities for needy students could have an effect in reducing high-risk sexual behaviour among students. Students whose parents are no longer alive are vulnerable to high-risk sexual behaviour and should be assisted with funds for survival on campuses. This could limit their sexual risk and improve their sexual health and wellbeing.

Our study also found that living with both parents was positively related to protective sexual behaviours. Individuals who lived with both of their parents had a higher likelihood of practising sexual abstinence. The finding holds after controlling for age and sex but not after controlling for lifestyle behaviours such as alcohol and drug use. This finding is consistent with a study that shows that adolescents and young adults living with neither parents have lower odds of delaying sex [13]. Some studies show that family structure is associated with sexual behaviour of adolescents and young adults [33, 42]. Nonetheless, children from single-parent households are significantly disadvantaged in terms of the parent’s time investment. The advantage of having two parents could be as a result of support from one of the parent if the other is at work. Moreover, young adults from two-parent households may have more family support available to them and this may reduce risky sexual behaviours.

### Study limitations

Even though this study advances the discourse on protective sexual behaviour of adolescents and young adults, the findings should be understood within its limitations. First, this is a cross-sectional study, as such, causality inference could not be drawn from it. The potential for social desirability bias could not be ruled out in self-reported sexual behaviour. Participants may have underreported their sexual activity and condom use because they are socially desirable. However, we tried to minimise this by having this discussion in safe spaces where young adults would be able to express themselves freely, and we also guaranteed confidentiality. We also ensured that males interviewed males and females interviewed females. Also, this study was conducted among university students who are generally more educated than adolescents and young adult’s population in Nigeria. Thus this study is not generalisable to Nigerian adolescent and young adult’s population. Also, due to our small sample size and the multiple categories created for sexual behaviours, we were unable to assess all categories of protective sexual behaviour. As such, future studies should use a larger sample, which will provide a large pool of young adults in each of the categories of protective sexual behaviours, thus, providing a better opportunity for results with higher precision.

## Conclusion

This study found that the majority of adolescents and young adults in Nigerian universities engage in protective sexual behaviours. Adequate family support and living with both parents are positively associated with protective sexual behaviour. The finding that about one-fifth of the respondents engage in high-risk sexual behaviour suggests that there is a need for behavioural change intervention that not only provides access to sexual health services but also empowers students who receive inadequate support from their family.

## Data Availability

Data analysed in this study will be made available by the corresponding author upon reasonable request.

## Abbreviations

HIV: Human immunodeficiency syndrome
STI: Sexually transmissible infections

## References

[1] Cort David A., Tu Hsin Fei (2018). Safety in stigmatizing? Instrumental stigma beliefs and protective sexual behavior in Sub-Saharan Africa. Social Science & Medicine.

[2] Blignaut Rénette J., Jacobs Joachim, Vergnani Tania (2015). Trends in HIV risk behaviour of incoming first-year students at a South African university: 2007–2012. SAHARA-J: Journal of Social Aspects of HIV/AIDS.

[3] Okafor I, Obi S (2005). Sexual risk behaviour among undergraduate students in Enugu, Nigeria. J Obstet Gynaecol.

[4] Okeke SR, Odelola JO (2018). Spatial and social settings as predictors of risky sexual behaviour among undergraduates in a Nigerian university. J Interprof Educ Pract.

[5] Perera UAP, Abeysena C (2018). Prevalence and associated factors of risky sexual behaviors among undergraduate students in state universities of Western Province in Sri Lanka: a descriptive cross sectional study. Reprod Health.

[6] Ševčíková A (2016). Girls’ and boys’ experience with teen sexting in early and late adolescence. J Adolesc.

[7] Fortenberry JD (2013). Puberty and adolescent sexuality. Horm Behav.

[8] Ruble DN. A phase model of transitions: cognitive and motivational consequences. In: Advances in experimental social psychology, vol. 26: Elsevier; 1994. p. 163–214.

[9] Sawyer SM, Azzopardi PS, Wickremarathne D, Patton GC (2018). The age of adolescence. Lancet Child Adolesc Health.

[10] UNAIDS (2017). Global HIV statistics: fact sheet July 2017.

[11] UNAIDS (2018). Miles to go—closing gaps, breaking barriers, righting injustices.

[12] World Health Organization (2014). Report on global sexually transmitted infection surveillance 2013.

[13] Odimegwu C, Somefun OD, Chisumpa VH. Regional differences in positive sexual behaviour among youth in sub-Saharan Africa. J Biosoc Sci. 2019;51(2):254-72. 10.1017/S002193201800010X 29633671

[14] Amo-Adjei J, Tuoyire DA (2018). Timing of sexual debut among unmarried youths aged 15–24 years in sub-Saharan Africa. J Biosoc Sci.

[15] Doyle AM, Mavedzenge SN, Plummer ML, Ross DA (2012). The sexual behaviour of adolescents in sub-Saharan Africa: patterns and trends from national surveys. Tropical Med Int Health.

[16] Somefun Oluwaseyi Dolapo, Odimegwu Clifford (2018). The protective role of family structure for adolescent development in sub-Saharan Africa. PLOS ONE.

[17] Mmari Kristin, Kalamar Amanda M., Brahmbhatt Heena, Venables Emilie (2016). The Influence of the Family on Adolescent Sexual Experience: A Comparison between Baltimore and Johannesburg. PLOS ONE.

[18] Pilgrim Nanlesta A., Ahmed Saifuddin, Gray Ronald H., Sekasanvu Joseph, Lutalo Tom, Nalugoda Fred, Serwadda David, Wawer Maria J. (2013). Family structure effects on early sexual debut among adolescent girls in Rakai, Uganda. Vulnerable Children and Youth Studies.

[19] Davis Erin Calhoun, Friel Lisa V. (2001). Adolescent Sexuality: Disentangling the Effects of Family Structure and Family Context. Journal of Marriage and Family.

[20] De Looze M, Constantine NA, Jerman P, Vermeulen-Smit E, ter Bogt T. Parent–adolescent sexual communication and its association with adolescent sexual behaviors: A nationally representative analysis in the Netherlands. J Sex Res. 2015;52(3):257-68. 10.1080/00224499.2013.858307 24512029

[21] Mmbaga Elia John, Leonard Frida, Leyna Germana Henry (2012). Incidence and Predictors of Adolescent’s Early Sexual Debut after Three Decades of HIV Interventions in Tanzania: A Time to Debut Analysis. PLoS ONE.

[22] Marteleto Letícia J., Cavanagh Shannon, Prickett Kate, Clark Shelley (2016). Instability in Parent-Child Coresidence and Adolescent Development in Urban South Africa. Studies in Family Planning.

[23] Adedimeji AA, Omololu FO, Odutolu O. HIV risk perception and constraints to protective behaviour among young slum dwellers in Ibadan, Nigeria. J Health Popul Nutr 2007;25(2):146. PMC2753996 17985816

[24] Ajayi AI, Nwokocha EE, Akpan W, Adeniyi OV, Goon DT. “It’s sweet without condom”: understanding risky sexual behaviour among Nigerian Female University students. Online J Health Allied Sci. 2018;16.

[25] Brian AJI, Umeononihu O, Echendu AD, Eke N. Sexual Behaviour among Students in a Tertiary Educational Institution in Southeast Nigeria. Advances in Reproductive Sciences. 2016;4(03):87.

[26] Enwereji E, Akubugwo E, Onwuka J, Ckikezie D (2017). Students’ sexual exposure in tertiary institutions: a case study of some universities in Abia state of Nigeria. Int J Community Med Public Health.

[27] Imaledo JA, Peter-Kio OB, Asuquo EO. Pattern of risky sexual behavior and associated factors among undergraduate students of the University of Port Harcourt, Rivers state, Nigeria. Pan Afr Med J. 2012;12. PMC3489398 23133697

[28] Ajayi AI, Ismail KO, Adeniyi OV, Akpan W (2018). Awareness and use of pre-exposure and postexposure prophylaxes among Nigerian university students: findings from a cross-sectional survey. Medicine.

[29] Benotsch EG, Snipes DJ, Martin AM (2013). Bull SS: sexting, substance use, and sexual risk behavior in young adults. J Adolesc Health.

[30] Snipes DJ, Benotsch EG (2013). High-risk cocktails and high-risk sex: examining the relation between alcohol mixed with energy drink consumption, sexual behavior, and drug use in college students. Addict Behav.

[31] Markham CM, Tortolero SR, Escobar-Chaves SL, Parcel GS, Harrist R, Addy RC (2003). Family connectedness and sexual risk-taking among urban youth attending alternative high schools. Perspect Sex Reprod Health.

[32] Odimegwu C, Adedini SA. Do family structure and poverty affect sexual risk behaviors of undergraduate students in Nigeria? Afr J Reprod Health. 2013;17(4):137-49. 24558790

[33] Peres CA, Rutherford G, Borges G, Galano E, Hudes ES, Hearst N (2008). Family structure and adolescent sexual behavior in a poor area of Sao Paulo, Brazil. J Adolesc Health.

[34] Ajayi AI, Nwokocha EE, Adeniyi OV, Ter Goon D, Akpan W. Unplanned pregnancy-risks and use of emergency contraception: a survey of two Nigerian Universities. BMC Health Serv Res. 2017;17(1):382. 10.1186/s12913-017-2328-7 PMC5455101 28577526

[35] Marks G, Crepaz N, Senterfitt JW, Janssen RS (2005). Meta-analysis of high-risk sexual behavior in persons aware and unaware they are infected with HIV in the United States: implications for HIV prevention programs. JAIDS J Acquir Immune Defic Syndr.

[36] Ajayi Anthony Idowu, Abioye Abdulazeez Olumide, Adeniyi Oladele Vincent, Akpan Wilson (2018). Concerns about contracting HIV, knowing partners’ HIV sero-status and discussion of HIV/STI with sexual partners as determinants of uptake of HIV testing. Journal of Biosocial Science.

[37] Stephenson R, Winter A, Elfstrom M (2013). Community environments shaping transactional sex among sexually active men in Malawi, Nigeria, and Tanzania. AIDS care.

[38] Tade O, Adekoya A (2012). Transactional sex and the ‘aristo’phenomenon in Nigerian universities. Hum Aff.

[39] Ajayi Anthony Idowu, Somefun Oluwaseyi Dolapo (2019). Transactional sex among Nigerian university students: The role of family structure and family support. PLOS ONE.

[40] MacPherson EE, Sadalaki J, Njoloma M, Nyongopa V, Nkhwazi L, Mwapasa V, Lalloo DG, Desmond N, Seeley J, Theobald S. Transactional sex and HIV: understanding the gendered structural drivers of HIV in fishing communities in southern Malawi. J Int AIDS Soc. 2012;15. 10.7448/IAS.15.3.17364 PMC3499929 22713352

[41] Jewkes R, Dunkle K, Nduna M, Shai NJ. Transactional sex and HIV incidence in a cohort of young women in the stepping stones trial. J AIDS and Clin Res. 2012;3(5).

[42] Ali MM, Ajilore O. Risky sexual behavior and african-american youth: What is the role of family structure? J Health Behav & Pub Health. 2011;1(1):30-40.

